# Gamma secretase activating protein promotes end-organ dysfunction after bacterial pneumonia

**DOI:** 10.1152/ajplung.00018.2023

**Published:** 2023-06-27

**Authors:** Meredith S. Gwin, Mikhail F. Alexeyev, Aron M. Geurts, Ji Young Lee, Chun Zhou, Xi-Ming Yang, Michael V. Cohen, James M. Downey, Robert A. Barrington, Domenico Spadafora, Jonathon P. Audia, Dara W. Frank, Sarah Voth, Viktoriya V. Pastukh, Jessica Bell, Linn Ayers, Dhananjay T. Tambe, Amy R. Nelson, Ron Balczon, Mike T. Lin, Troy Stevens

**Affiliations:** ^1^Department of Physiology and Cell Biology, Center for Lung Biology, University of South Alabama, Mobile, Alabama, United States; ^2^Department of Physiology, Medical College of Wisconsin, Milwaukee, Wisconsin, United States; ^3^Department of Microbiology and Immunology, Center for Lung Biology, University of South Alabama, Mobile, Alabama, United States; ^4^Department of Microbiology and Immunology, Medical College of Wisconsin, Milwaukee, Wisconsin, United States; ^5^Department of Cell Biology and Physiology, Edward Via College of Osteopathic Medicine, Monroe, Louisiana, United States; ^6^Department of Mechanical, Aerospace, and Biomedical Engineering, Center for Lung Biology, University of South Alabama, Mobile, Alabama, United States; ^7^Department of Biochemistry and Molecular Biology, Center for Lung Biology, University of South Alabama, Mobile, Alabama, United States

**Keywords:** acute lung injury, beta amyloid, long-term potentiation, myocardial infarction, pneumonia

## Abstract

Pneumonia elicits the production of cytotoxic beta amyloid (Aβ) that contributes to end-organ dysfunction, yet the mechanism(s) linking infection to activation of the amyloidogenic pathway that produces cytotoxic Aβ is unknown. Here, we tested the hypothesis that gamma-secretase activating protein (GSAP), which contributes to the amyloidogenic pathway in the brain, promotes end-organ dysfunction following bacterial pneumonia. First-in-kind *Gsap* knockout rats were generated. Wild-type and knockout rats possessed similar body weights, organ weights, circulating blood cell counts, arterial blood gases, and cardiac indices at baseline. Intratracheal *Pseudomonas aeruginosa* infection caused acute lung injury and a hyperdynamic circulatory state. Whereas infection led to arterial hypoxemia in wild-type rats, the alveolar-capillary barrier integrity was preserved in *Gsap* knockout rats. Infection potentiated myocardial infarction following ischemia-reperfusion injury, and this potentiation was abolished in knockout rats. In the hippocampus, GSAP contributed to both pre- and postsynaptic neurotransmission, increasing the presynaptic action potential recruitment, decreasing neurotransmitter release probability, decreasing the postsynaptic response, and preventing postsynaptic hyperexcitability, resulting in greater early long-term potentiation but reduced late long-term potentiation. Infection abolished early and late long-term potentiation in wild-type rats, whereas the late long-term potentiation was partially preserved in *Gsap* knockout rats. Furthermore, hippocampi from knockout rats, and both the wild-type and knockout rats following infection, exhibited a GSAP-dependent increase in neurotransmitter release probability and postsynaptic hyperexcitability. These results elucidate an unappreciated role for GSAP in innate immunity and highlight the contribution of GSAP to end-organ dysfunction during infection.

**NEW & NOTEWORTHY** Pneumonia is a common cause of end-organ dysfunction, both during and in the aftermath of infection. In particular, pneumonia is a common cause of lung injury, increased risk of myocardial infarction, and neurocognitive dysfunction, although the mechanisms responsible for such increased risk are unknown. Here, we reveal that gamma-secretase activating protein, which contributes to the amyloidogenic pathway, is important for end-organ dysfunction following infection.

## Graphical abstract

**Figure:**
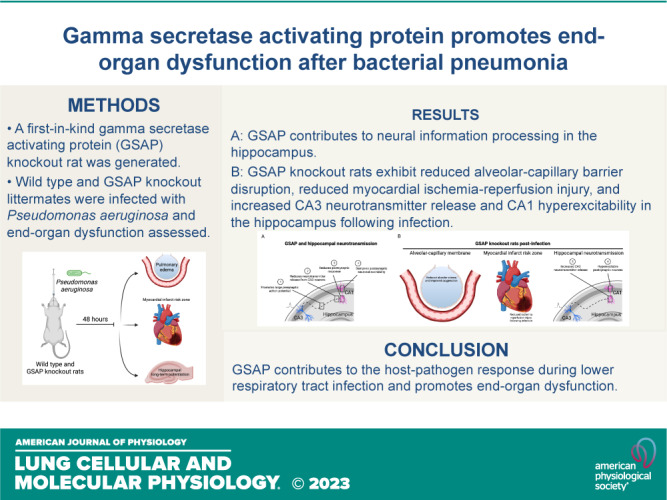

## INTRODUCTION

Gamma secretase activating protein (GSAP) is a recently described subunit of the gamma (γ)-secretase complex (1). GSAP is expressed as a 98-kDa holoprotein, and the holoprotein is cleaved by caspase-3 or other enzymes to generate a 16-kDa product (2, 3). The 16-kDa GSAP interacts with the adaptor protein Fe65 and presenilin-1, which are components of the gamma-secretase enzyme complex, in the mitochondria-associated membrane, where it also binds to amyloid precursor protein (4, 5). GSAP thus localizes the gamma-secretase enzyme complex with amyloid precursor protein important to produce beta-amyloid (Aβ) variants, including Aβ_40_ and Aβ_42_ (4, 6, 7). However, GSAP has an additional subcellular function(s). It interacts with multiple proteins and fulfills an essential signal transduction function, including regulation of mitochondrial oxygen consumption (5). *Gsap* deletion in cells and mice attenuates the production of both Aβ_40_ and Aβ_42_ and also increases cellular respiration important for cell survival. Cognitive function is improved in *Gsap* knockout animal models of Alzheimer’s disease, consistent with the amyloid hypothesis of neurodegeneration (1, 2, 4, 6, 8). Although GSAP is best known for its influence on Aβ production in the brain, its contributions to subcellular signal transduction and protein trafficking promotes cell survival, illustrating its physiological role extends beyond the production of Aβ (5). Yet, the function(s) of GSAP outside of the brain have not been addressed, and thus, its physiological relevance remains incompletely understood.

GSAP is ubiquitously expressed, including in the lungs and heart (9), and Aβ has diverse physiological functions within and outside the brain (10). For example, Aβ plays a pivotal role in the host’s response to infection. It can have antimicrobial properties (11–15) and it can also be pathogenic to the host (16–18). Aβ is increased in the bronchoalveolar lavage fluid, blood, and/or cerebrospinal fluid during infection (16–18), and infection is potentially an adverse effect of Aβ-lowering medical therapy (19). Aβ contributes to the pathogenesis of ischemic heart disease (20), it can activate platelets (21), and it can injure the lung (22, 23). Although these studies suggest a central role for Aβ in the innate immune response, mechanisms contributing to the increase in Aβ during infection are unresolved.

Pneumonia is a common cause of acute respiratory failure, and it substantially increases the risk of morbidity and mortality during, and in the aftermath, of infection (24). Cardiovascular disease and neurocognitive dysfunction, including dementia, impairments in learning and memory, depression, anxiety, and posttraumatic stress disorder, are prevalent among survivors of critical illness (25–27). Approximately one-third of patients who survive their critical illness exhibit cognitive impairment similar to patients with mild Alzheimer’s disease, which is characterized by impaired neural information processing in the hippocampus leading to reductions in learning and memory (25). The mechanism(s) of this incident cognitive impairment is unknown, however, we found cytotoxic forms of Aβ in the airways, blood, and cerebrospinal fluid of intensive care unit patients and animal subjects with nosocomial pneumonia (16–18, 28). Based on these studies, and the role that GSAP plays in regulating Aβ production in the brain, we sought to determine whether GSAP contributes to the host-pathogen response during infection, mediating end-organ dysfunction that is a consequence of pneumonia.

## MATERIALS AND METHODS

### Ethical Use of Rats for Research

Rats were group-housed in microisolation caging with enrichment, according to the established guidelines for the care and use of laboratory rats. Rooms were on a 12:12-h light-dark cycle, and the temperature was controlled. All animal procedures performed in this study were reviewed and approved by the University of South Alabama Institutional Animal Care and Use Committee. The genotype of the animals that were studied was blinded to investigators, when possible.

### Generation of GSAP Knockout Rats

*Gsap* knockout rats were generated at the Medical College of Wisconsin and shipped to the University of South Alabama for this study. Briefly, a single guide RNA targeting the sequence GTCCTTTGCAGTCCCTGCCG within exon 16 of *Gsap* (GenBank NM_001395028.2) was mixed with Cas9 (*S. pyogenes*) protein (QB3 MacroLab, UC Berkeley) and injected into the pronucleus of Sprague Dawley (Crl:SD, Charles River Laboratories) embryos. Among the offspring, a mutant founder was identified harboring a 5-bp deletion (rn72: chr4:13,849,488-13,849,492) mutation inducing a frame-shift in the *Gsap* coding sequence predicted to truncate the normal 846 amino acid GSAP protein (GenPept NP_001381957.1) after 382 amino acids. The founder was backcrossed to the parental Crl:SD strain to establish a breeding colony and was designated SD-*Gsap^em2Mcwi^* (*Gsap* KO).

Once rats were received at the University of South Alabama, they underwent a 30-day quarantine, after which time, pathogen testing revealed they were all pathogen-free. Rats were bred in house. *Gsap* KO rats were genotyped using an equimolar mix of primers rGSAP4delIntF ( CGAGTCCTTTGCAGTCCG), rGSAP4delR ( GAAGCACCAAGGGAGACTATG), rGSAP34F ( AGGCTACTTTGTGGCTGTTTATTC), and rGSAPintR3 ( GAGGGACCCCGGCAGGG). PCR conditions were: initial denaturation of 1 min at 95°C followed by 35 cycles of 95°C for 10 s, 55.7°C for 20 s, and 72°C for 40 s, followed by a 5-min hold at 7°C and an infinite hold at 10°C. With this strategy, the DNA of wild-type rats produces an amplicon of 268 bp, the DNA of knockout rats produces an amplicon of 318 bp, and the DNA from heterozygous rats produces amplicons of both sizes.

### Pseudomonas aeruginosa

ExoY is a *P. aeruginosa* type three secretion system effector. It is a promiscuous nucleotidyl cyclase that preferentially generates canonical (cGMP and cAMP) and noncanonical (cUMP and cCMP) cNMPs when injected into host cells, including the lung microvasculature (29–33). The ExoY^+^ (PA103Δ*exoUexoT*::Tc/pUCP-*exoY*) mutant was generated by Dr. Dara Frank. ExoY^+^ was used in this study because it, like clinical strains that utilize ExoY in their virulence arsenal, elicits production of cytotoxic Aβ and tau variants in the lung that disseminate through the circulation to peripheral organs, including the heart and brain.

The ExoY effector is expressed in ExoY^+^ via a plasmid. Because of this, the bacteria are maintained in selection media consisting of Luria Broth (Miller’s), glycerol, and carbenicillin (400 µg/mL) and kept in a −80°C freezer. The day before the planned experiment, bacteria were taken from previously frozen stocks, struck on Vogel-Bonner agar plates supplemented with 400 µg/mL of carbenicillin, and grown overnight at 37°C. On the day of the experiment the bacteria are resuspended in PBS to an optical density (OD_540_) allowing for calculation of a final concentration of 10^7^–10^8^ bacteria (depending on the experiment conducted) in 250 µL of PBS.

### Bacterial Culture and Intratracheal Inoculation

ExoY^+^ bacteria were grown overnight on Vogel-Bonner medium containing 400 µg/mL of carbenicillin (SCBT CAS 4800-94-6) at 37°C. The morning of the inoculation the bacteria were resuspended in PBS until an OD_540_ of 0.500 (10^8^ bacteria in 250 µL PBS) was reached, as determined by a spectrophotometer (Thermo Fisher AquaMate UV/Vis). Rats were anesthetized using isoflurane gas (1%–3%) and a surgical plane was achieved, as noted by the lack of toe and tail pinch reflex. The skin and muscle over the trachea were separated using a No. 11 scalpel, and a 4-0 suture was placed to retract the muscle and allow for an unobstructed view of the trachea. Bacteria were thoroughly vortexed to ensure single-cell suspension and collected into a 1 mL tuberculin syringe. The surgical table was adjusted to an ∼70^°^ angle, the needle was inserted into the trachea, and the bacteria were instilled slowly over 5 min using a circular motion to ensure the bacteria were distributed evenly. The skin was sutured, and the rats were given Ethiqua (buprenorphine extended-release injectable suspension; 0.65 mg/kg body wt) via a single subcutaneous injection for pain management during recovery. Terminal experiments were conducted at 48 h postinfection, unless otherwise specified.

### Measurement of Bacteria in the Lung, Blood, and Spleen

Wild-type and knockout animals were infected with *P. aeruginosa*, and at 48 h the lung, blood, and spleen were collected. The lung lobes were dissected and weighed, and the weights were recorded. The lung lobes were then minced into small pieces using a razor blade. Sterile PBS was added to the lung pieces which were then homogenized into a pipette-able slurry. Two hundred microliter of the slurry was weighed and recorded. Of the remaining slurry, 100 µL was pipetted onto two pseudomonas isolation agar (PIA) plates, and the slurry was distributed across the plate using sterile glass beads. The beads were removed and the plate was allowed to dry in a 37°C bacterial incubator. The slurry was serially diluted, and the dilutions were plated. A drip plate including multiple dilutions was made for easier visualization of the different dilutions. Plates were allowed to grow for 24 h, at which point images were taken and bacterial colonies were counted. Colonies were allowed to grow for 24 h more to ensure all colonies were counted.

### Measurement of Arterial Blood Gases and Collection of Plasma

Rats were anesthetized via isoflurane (1%–3%), and a transperitoneal approach was utilized to isolate the abdominal aorta and separate it from the inferior vena cava. The abdominal aorta was catheterized using a 24 g catheter, blood was drawn into a 1 mL syringe for arterial blood gas measurements (Stat Profile Prime^+^ Critical Care Analyzer, Nova Biomedical), and then blood was drawn into a 10 mL syringe for separation into plasma (lithium heparin), an electrolyte panel (heparin), and analysis of complete blood counts with differential (EDTA). To isolate the plasma fraction, whole blood was centrifuged at 200 *g* at 4°C for 10 min (Eppendorf 5910 R). Once separated, the plasma was removed and stored at −80°C.

### Organ Harvesting

Male and female *Gsap* knockout rats and controls were anesthetized using isoflurane gas (1%–3%). Once a surgical plane was achieved, as defined by the absence of a withdrawal reflex following toe and tail pinch, the rats were placed on their backs with their nose connected to the isoflurane tank via plastic tubing. Hair was removed from the abdominal and chest areas using hair clippers and hair removal paste. A midline incision was made using a No. 10 scalpel. Organs including spleen, liver, brain, lungs, heart, and thymus were isolated and removed. Organ wet weights were measured and recorded. Organs were then allowed to dry over 7 days. After 7 days, dry weights were measured and the wet-to-dry weight ratio was calculated. In separate experiments, the organs were removed and placed into conical tubes, flash frozen with liquid nitrogen, and stored at −80°C.

### Echocardiography

A Vevo 3100 (VisualSonics, Toronto, ON, Canada) with a 30 MHz transducer (MX550D) was used to evaluate cardiac and pulmonary function. Spontaneously breathing rats were anesthetized with isoflurane (∼1.5%, titrated as needed) in a 1:1 O_2_–air admixture. Heart rate, electrocardiogram, and respiration were continuously recorded using the sensor-embedded exam pad, whereas cardiac and pulmonary ultrasound parameters were assessed, as described in detail previously (22).

### Isolated Perfused Lung

Wild-type and knockout rats were anesthetized using pentobarbital sodium (65 mg/kg body wt). Once a surgical plane was achieved, as defined by the absence of a withdrawal reflex following toe and tail pinch, rats were intubated and ventilated, a sternotomy was performed, and pulmonary artery and left ventricle/atrium catheters were placed. Heart and lungs were removed en bloc and suspended in a humidified chamber, where mechanical ventilation and flow were established. Rat lungs were perfused with buffer (in mmol/L: 119.0 NaCl, 4.7 KCl, 1.17 MgSO_4_, 1.18 KH_2_PO_4_, 23 NaHCO_3_, and 5.5 glucose) containing 4% bovine serum albumin and physiological (2.2 mmol/L) CaCl_2_, plus 6% autologous whole blood. After an initial isogravimetric period a baseline filtration coefficient (*K*_f_) was measured. Filtration coefficient was calculated as the rate of weight gain obtained 13–15 min following a 10 cmH_2_O increase in pulmonary venous pressure, normalized per 100 g of the predicted wet lung weight. Pulmonary artery and venous pressures and lung weight were measured continuously, and double occlusion pressure was measured under stop-flow conditions. Following the baseline *K*_f_, bacteria were instilled into the trachea at a concentration of 1 × 10^8^ CFU. Four hours later, *K*_f_ was measured again to assess the impact of airway infection on alveolar-capillary permeability.

### Bronchoalveolar Lavage Fluid Collection and Analysis

Rats were infected with ExoY^+^ (10^8^) and monitored for over 48 h. Control and infected rats were then anesthetized with isoflurane gas (1%–3%) and once a surgical plane was established, as determined by a negative toe and tail pinch reflex, the trachea was cannulated, and a sternotomy was performed, removing the heart and lungs en bloc. The cannula was connected to tubing attached to a 60 mL syringe that was adjusted to provide 25 cmH_2_O filling pressure. PBS was allowed to flow into the lungs using the force of gravity until full (∼10 mL), at which point the tubing was disconnected and a syringe connected directly to the tracheal cannula to remove the PBS. This process was repeated three times to collect ∼30 mL of bronchoalveolar lavage fluid (BALF). BALF was quickly vortexed, and 1 mL was transferred into a separate tube for cell counting using a Countess 3 Cell Counter (Thermo Fisher) and bacterial cell counting. The remaining BALF was centrifuged at 500 *g* for 5–10 min. The supernatant was collected and sterilized via a 0.2 µm filter and frozen at −80° for later use. The remaining cell pellet was frozen at −80°C in full DMEM containing 20% FBS and 14% DMSO for analysis at a later date.

### Measurement of Cytokines

Plasma collected from control and 48-h ExoY^+^ (10^8^)-infected WT and KO rats was analyzed for inflammatory cytokines according to manufacturer instructions (BioLegend). The rat inflammation panel (13-plex with V-bottom Plate; No. 740401) was used for these studies.

### Measurement of Aβ

Plasma was collected from control and 48-h ExoY^+^ (10^8^)-infected WT and KO rats and was analyzed for three different species of beta-amyloid (1–38, 1–40, and 1–42) according to manufacturer instructions [Meso Scale Discovery V-PLEX Aβ Peptide Panel 1 (4G8) Kit No. K15199E-1].

### Flow Cytometry

WT and KO rats were infected with ExoY^+^ (10^8^) over 48 h. Control and infected rats were then anesthetized with isoflurane gas (1%–3%) and once a surgical plane was established as determined by a negative toe and tail pinch reflex, the trachea was cannulated, and a sternotomy was performed, removing the heart and lungs en bloc. The cannula was connected to tubing attached to a 60 mL syringe that was adjusted to provide 25 cmH_2_O filling pressure. PBS was allowed to flow into the lungs using the force of gravity until full (∼10 mL), at which point the tubing was disconnected and a syringe connected directly to the tracheal cannula to remove the PBS. This process was repeated three times to collect ∼30 mL of bronchoalveolar lavage fluid (BALF). BALF was quickly vortexed, and 1 mL was transferred into a separate tube for cell counting using a cell countess. The remaining BALF was placed on ice and centrifuged at 500 *g* for 10 min. Supernatant was collected, filter-sterilized with a 0.2 µm filter, and frozen at −20°C. The remaining cell pellet was resuspended in 1 mL PBS for use the same day or frozen at −80°C in full DMEM containing 20% FBS and 14% DMSO for analysis later.

Frozen cells were thawed quickly at 37°C and put into a flow tube containing 3 mL of DMEM. Cells were pelleted at 1,400 rpm for 7 min. DMEM was removed, and the cell pellet was resuspended in PBS to a final 10^6^ cell count/mL of PBS. Cells were centrifuged at 1,300 rpm for 6 min and PBS was removed. Cells were resuspended in an antibody cocktail containing PE/Dazzle 594 anti-rat CD45 (BioLegend No. 202224; 0.2 mg/mL), PE/Cyanine7 anti-rat CD11b/c (BioLegend No. 201818; 0.2 mg/mL), mouse anti-rat CD43:RPE (Bio-Rad No. MCA54PE; 10 µL/1 million cells), mouse anti-rat CD68 (Bio-Rad No. MCA341R; 1 mg/mL), BV605 mouse anti-rat RP-1 (BDBiosciences No. 743055; 0.2 mg/mL), and mouse anti-rat His48 (ThermoFisher No. 11-0570-82; 0.5 mg/mL). BALF cells were incubated with the antibody cocktail away from light and on ice for 30 min. Three milliliters of PBS were added to the cells and antibody cocktail, quickly vortexed, and centrifuged at 1,480 rpm for 7 min. PBS and antibody cocktail were aspirated, and the remaining cells were resuspended in fresh PBS for flow cytometric analysis. Fluorescence-activated flow cytometry was performed using a CytoFLEX S flow cytometer (Beckman Coulter) to sort cells, and the analysis was performed using FlowJo v.10.8 Software (BD Life Sciences).

### Myocardial Infarction in the Open Chest Rat

Rats were infected with ExoY^+^ (10^7^) over 48 h, as described earlier. For these studies a lower bacterial concentration was used to ensure animals survived to the time of the ischemia-reperfusion injury and to ensure they could tolerate the anesthesia and surgery. At 48 h, rats were anesthetized via intraperitoneal delivery of pentobarbital sodium (65 mg/kg body wt) to a surgical plane, as determined by the absence of a toe pinch reflex. The right jugular vein was then catheterized and anesthesia was maintained throughout the experimental period by intravenous delivery of pentobarbital sodium. The left carotid artery was catheterized for measurement of blood pressure. A snare was placed around the left anterior descending branch of the left coronary artery, and the snare was tightened to allow for ischemia for 30 min. After 30 min, the snare was removed to allow for 2 h of reperfusion.

After the 2-h period, the heart was removed and mounted on a Langendorff apparatus and perfused at 100 mmHg with normal saline through the aortic root. After 2 min of reperfusion, the coronary artery branch was reoccluded, and 2–9 µm fluorescent beads were infused into the heart. The heart was flash frozen in liquid nitrogen, cut into 2 mm transverse slices, and incubated for 8–10 min in triphenyltetrazolium chloride. The risk area was visualized as myocardium without fluorescent microspheres. All slices that included any tissue without fluorescent microspheres (the ischemic zones) were arranged in order from the base to the apex and sandwiched together between 2 glass plates spaced 2 mm apart. The border between the ischemic and normal tissue for each slice was traced with a fine tip marker on the top glass plate under a UV light. The mounted slices were then photographed on a blue background. Using an image editor, the ischemic zone for each slice in the resulting image was traced, copied, and pasted on a blue background color to form a new image having only the risk zones. Using the color threshold feature of the ImageJ program, the area of just the infarcts and then the entire risk zones were both measured in the risk zone image. The percent infarction of the risk zone could then be calculated for the entire heart. The same color threshold settings for the infarct and for the entire risk zone were used for all the hearts.

### Hippocampi Isolation and Long-Term Potentiation

Immediately after the harvest of peripheral organs, rat brain was obtained and kept in an ice-cold slushy sucrose-artificial cerebrospinal fluid (in mM): 70 sucrose, 80 NaCl, 2.5 KCl, 21.4 NaHCO_3_, 1.25 NaH_2_PO_4_, 0.5 CaCl_2_, 7 MgCl_2_, 1.3 ascorbic acid, and 20 glucose. After 1 min of incubation, the hippocampi were dissected out, placed onto an agar block, and sliced at 300 µm using a Lecia VT1200s (Leica Instruments). Transverse hippocampal slices were placed into a holding chamber containing regular artificial cerebrospinal fluid (aCSF; in mM): 125 NaCl, 2.5 KCl, 21.5 NaHCO_3_, 1.25 NaH_2_PO_4_, 2.0 CaCl_2_, 1.0 MgCl_2_, and 15 glucose. Slices were incubated for 30 min at 35°C and then for >1 h at room temperature before they were transferred to an upright Leica microscope for field potential recording. All solutions were constantly equilibrated with carbogen (95% O_2_ and 5% CO_2_).

For hippocampal field potential recordings, hippocampal CA3 region was severed, and the brain slice was transferred to the recording chamber. Brain slices were superfused with aCSF at a flow rate of 1 mL/min at room temperature. The perfusing aCSF contained SR95531 (2 µM) and CGP55845 (1 µM) to block inhibitory synaptic transmission. Brain slices were held in place with a harp slice anchor (Warner Instruments), and stimulating and recording glass electrodes were placed in the CA1 stratum radiatum, ∼150 µm away from the somata, to stimulate the Schaffer collateral pathway. Stimulation was performed using an ISO-Flex stimulus isolation unit (A.M.P.I.), and recording was obtained using an EPC10 amplifier (HEKA Elektronik). Analog signals were further amplified ×10 using an Axopatch amplifier (Axon Instrument) and digitized/filtered either at 20 kHz/5 kHz (for field and paired-pulse ratio recordings) or 100 kHz/25 kHz (for input-output function recordings) using the Patchmaster software (HEKA Elektronik).

At the beginning of recording for each brain slice, an input-output function was determined, and the excitatory postsynaptic potential amplitude was set at ∼30% of maximum amplitude, followed by a series of paired-pulse ratio recordings, and then the field long-term potentiation (LTP). Paired-pulse stimulations, recorded every 10 s, were performed at 25-, 50-, 100-, 200-, and 400-ms interstimulus intervals, and five traces of each interval were averaged. For LTP studies, field excitatory postsynaptic potentials were evoked and recorded every 20 s, and a stable baseline of >10 min was established before a theta-burst stimulation consisting of three sweeps (a single burst consists of five stimuli delivered at 100 Hz and 10 bursts delivered at 5 Hz per sweep) was delivered at 30-s intervals to induce synaptic potentiation. LTP was calculated from field potentials recorded at 55–60 min post the theta-burst stimulation and normalized to the baseline field potentials before the theta-burst stimulation.

Offline data analyses of electrophysiological recordings were performed using custom macros written in Igor Pro (WaveMetrics). As a general rule for this study, field excitatory postsynaptic potential amplitude was determined from the peak, and the slope was measured between 10% and 50% of the rising phase. The time-to-population spike was determined from the trace of the strongest electrical stimulus (i.e., 100 µA), and the groove was set as the lowest point (closest to the 0 baseline) between the two field excitatory postsynaptic peaks. LTP data were binned at 3-min intervals to generate the summary field excitatory postsynaptic potential plot. Data are expressed as means ± standard deviation or standard error of the mean and statistically compared as specified. Figures were prepared in Igor Pro and/or Prism (GraphPad); data tabulation was done using Excel (Microsoft).

## RESULTS

### GSAP Contributes to the Lung’s Innate Immune Response in Pneumonia

**Figure 1.**
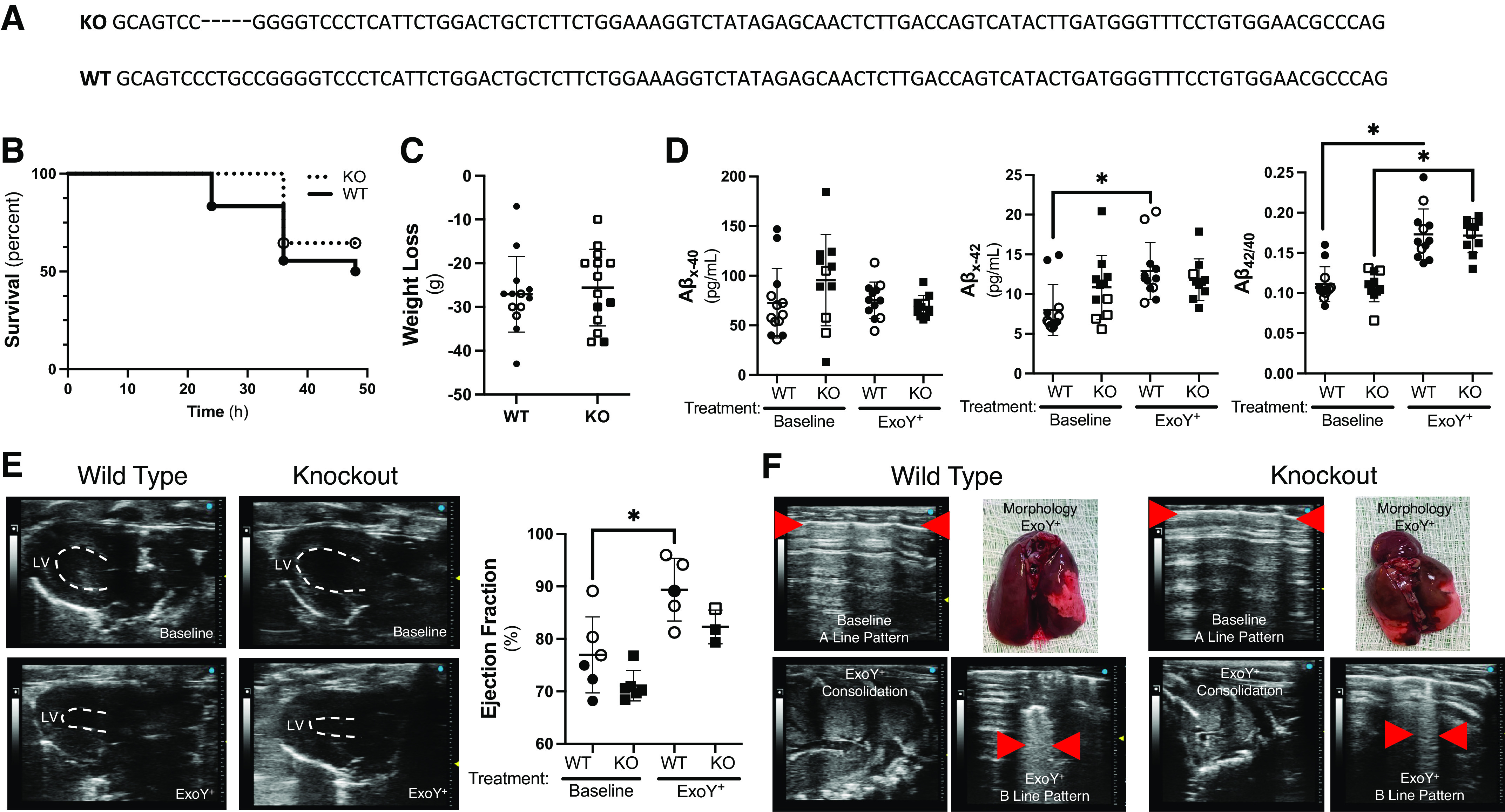
Wild-type and *Gsap* knockout rats exhibit similar infection-induced hemodynamic responses. *A:* schematic showing the 5 bp deletion in exon 16 of the *Gsap* gene produced using CRISPR/Cas9 technology. *B*: wild-type (WT; *n* = 18) and knockout (KO; *n* = 31) rats were infected with ExoY^+^ and the percentage of animals that survived up to the point of the terminal experiment at 48 h postinfection was measured. Eighty-three percent of wild-type rats survived 24 h postinfection, whereas 50% of the rats survived to 48 h postinfection. One hundred percent of the knockout rats survived 24 h postinfection, whereas 65% of the rats survived to 48 h postinfection (*P* = ns between WT and KO rats). *C*: wild-type (*n* = 13) and knockout (*n* = 14) rats were weighed before infection and 48 h postinfection. Rats lost ∼25 g body weight 48 h postinfection. There was no significant difference in weight loss between the two groups (two-tailed Welch’s *t* test, *P* = ns). *D*: Aβ_x-40_ (Aβ_40_) was measured in rats at baseline and 48 h following infection. There was no difference in the baseline circulating Aβ_40_ concentrations (post hoc *P* = ns; main effect for genotype, *P* = ns, and for infection, *P* = ns; *n* = 4–6). Aβ_x-42_ (Aβ_42_) was measured in rats at baseline and 48 h following infection. There was no difference in the baseline circulating Aβ_42_ concentrations (main effect for genotype, *P* = ns, and for infection, *P* = ns; post hoc, *P* = ns), yet the Aβ_42_ concentration was increased in wild type but not in the knockout rats 48 h postinfection (main effect for infection, *P* = 0.0337; post hoc, *P* = 0.0069; *n* = 4–6). The Aβ_42/40_ ratio was not different among the animal genotypes, but it was increased in both wild-type (*P* < 0.0001) and knockout (*P* < 0.0001) rats following infection (main effect for genotype, *P* = ns, and for infection, *P* ≤ 0.0001). *E*: cardiac ultrasound was assessed at baseline and 48 h postinfection. Ejection fraction was significantly increased postinfection in WT (main effect for genotype, *P* = 0.0444, and for infection, *P* = 0.0021; post hoc, *P* = 0.0365; *n* = 5 or 6) rats. *F*: pulmonary ultrasonography revealed consolidation and B lines (red arrowheads) in both the wild-type and knockout rats, postinfection. Lung morphology showed a heterogeneous pattern of lung injury indicative of bilobar pneumonia. Statistics were done using two-way ANOVA with a Tukey’s multiple comparisons test unless otherwise stated. Summary data are reported as means ± standard deviation. Open symbols reflect female subjects and closed symbols indicate male subjects. GSAP, gamma-secretase activating protein. *Statistically significant difference.

**Figure 2.**
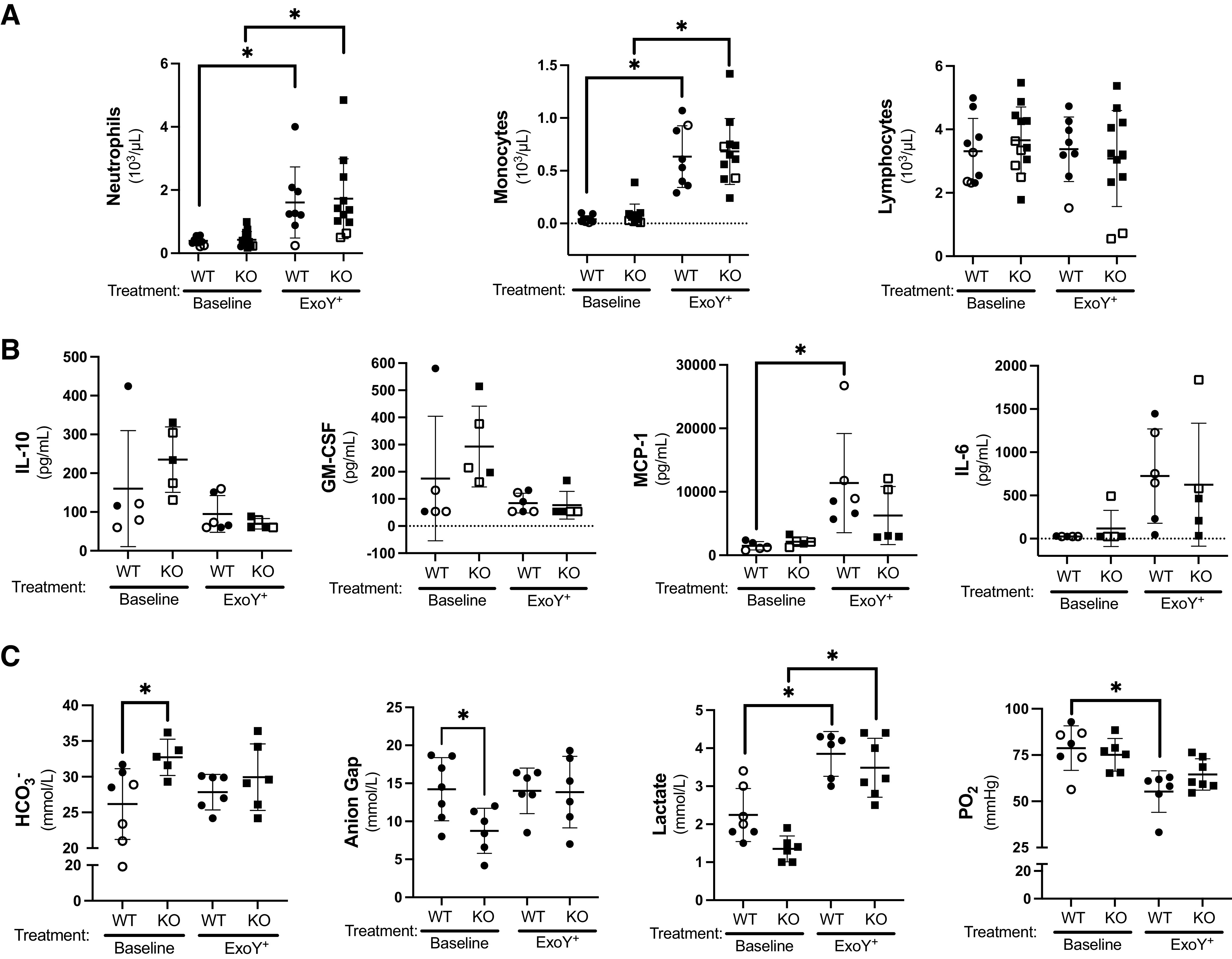
GSAP contributes to innate immunity and alveolar-capillary barrier integrity. Blood was collected from the abdominal aorta of control and ExoY^+^-infected WT and KO rats and a complete blood count with differential, a cytokine immunoassay in plasma, and an electrolyte panel were analyzed. *A*: WT and KO rats exhibited neutrophilia (main effect for genotype, *P* = ns, and for infection, *P* ≤ 0.0002; post hoc, WT, *P* = 0.037 and KO, *P* = 0.0025) and monocytosis (main effect for genotype, *P* = ns, and for infection, *P* ≤ 0.0001; post hoc WT, *P* < 0.0001 and KO, *P* < 0.0001; WT, *n* = 8–10 and KO, *n* = 11 or 12), whereas lymphocyte count remained unchanged (main effect for genotype, *P* = ns, and for infection, *P* = ns; post hoc, *P* = ns), after infection. *B*: the proinflammatory cytokine MCP-1 was increased following infection in the WT rats (main effect for genotype, *P* = 0.0023, and for infection, *P* = 0.0411; post hoc *P* = 0.0096; *n* = 5 or 6). *C*: KO rats had an increase in the bicarbonate concentration (main effect for genotype, *P* = 0.0280, and for infection *P* = ns; post hoc *P* = 0.0411) and a decrease in the anion gap (main effect for genotype, *P* = ns, and for infection, *P* = ns; post hoc, *P* = 0.0317) when compared with the WT controls at baseline (*n* = 5–7). After infection, lactate was increased in both WT (*P* = 0.0006) and KO rats (*P* < 0.0001; main effect for genotype, *P* = ns, and for infection, *P* ≤ 0.0001), whereas only WT rats exhibited a significant decrease in arterial oxygenation (main effect for genotype, *P* = ns, and for infection, *P* = 0.0016; post hoc $\text{P}{\text{a}}_{{\text{O}}_{2}}$, *P* = 0.0029; *n* = 5–7). Statistics were determined by two-way ANOVA with Tukey’s multiple comparisons test. Summary data are reported as means ± standard deviation. Open symbols reflect female subjects and closed symbols indicate male subjects. GSAP, gamma-secretase activating protein; KO, knockout; MCP, monocyte chemoattractant protein; WT, wild type. *Statistically significant difference.

We generated a first-in-kind *Gsap* knockout rat using CRISPR-Cas9 gene editing (Fig. 1*A*). A 5-nucleotide deletion was generated in exon 16 and was confirmed by genomic sequencing. The deletion resulted in a frameshift and premature termination of protein synthesis. Breeding of rats heterozygous for the *Gsap* allele over 27 pairings resulted in 626 progenies. Three hundred and fifteen of these pups were male and 311 were female, distributed among wild type (28%), knockout (25%), and heterozygote (46%) genotypes according to the expected Mendelian distribution. Body weights (Supplemental Fig. S1), organ sizes (Supplemental Table S1), complete blood cell counts (Supplemental Table S2), and electrolytes (Fig. 2 and Supplemental Fig. S2) were not significantly different among wild-type and *Gsap* knockout littermates under baseline conditions. All Supplemental material is available at https://doi.org/10.6084/m9.figshare.23304029.v2.

To examine whether GSAP contributes to the innate immune response following infection, *P. aeruginosa* (PA103Δ*exoUexoT*::Tc/pUCP-*exoY*; ExoY^+^) was introduced by intratracheal inoculation into wild-type and *Gsap* knockout rats (10^8^ CFU; ∼LD_50_). Fifty percent of wild-type rats survived 48 h postinfection, whereas 65% of the knockout rats survived to this time point (Fig. 1*B*). Both the wild-type and knockout rats lost ∼25 g body weight (10%–15%) 48 h postinfection (Fig. 1*C*), and hematocrit and hemoglobin were increased, consistent with volume depletion (Supplemental Fig. S3). Whereas circulating Aβ_40_ and Aβ_42_ concentrations were similar among the rats at baseline, infection increased Aβ_42_ (∼2-fold) in the wild type but not in the knockout rats (Fig. 1*D*). Nonetheless, the Aβ_42/40_ ratio increased in the plasma of both wild-type and knockout rats, postinfection, suggesting that GSAP is not a principal determinant of the circulating amyloid pool. Cardiopulmonary ultrasonography revealed a hyperdynamic circulatory state (Fig. 1*E*). Hyperechoic vertical B lines characteristic of lung edema and consolidation were apparent in both groups, and gross morphological assessment showed a heterogenous pattern of lung injury (Fig. 1*F*). The cardiopulmonary response to infection was therefore generally similar among wild-type and *Gsap* knockout rats. Lung, blood, and spleen were tested for the presence of bacteria postinfection. A significant bacterial burden was detected in the lung from both wild-type and knockout rats, but bacteria were not found in either the blood or spleen (data not shown).

Complete blood cell counts with differential revealed no difference in circulating platelets, red blood cells, or total white blood cell counts among genotypes under basal conditions. The percentage of circulating neutrophils and monocytes was increased, and the percentage of circulating lymphocytes was decreased, following infection (Supplemental Fig. S3). Neutrophilia and monocytosis were prominent after infection whereas total lymphocyte counts were unchanged (Fig. 2*A*). Two anti-inflammatory cytokines, IL-10 and GM-CSF trended toward higher values in *Gsap* knockout rats at baseline and were significantly reduced following infection. Two proinflammatory cytokines, MCP-1 and IL-6, were increased in wild-type rats following infection (Fig. 2*B*). Bicarbonate was elevated and lactate trended toward lower values in *Gsap* knockout rats at baseline, which lowered the anion gap, yet after infection, anion gap fell within the normal range in both wild-type and knockout rats (Fig. 2*C*). Infection increased lactate and impaired oxygenation; it was notable that oxygenation was better in the knockout rats, indicating improved alveolar-capillary barrier integrity (Fig. 2*C* and Supplemental Fig. S2).

**Figure 3.**
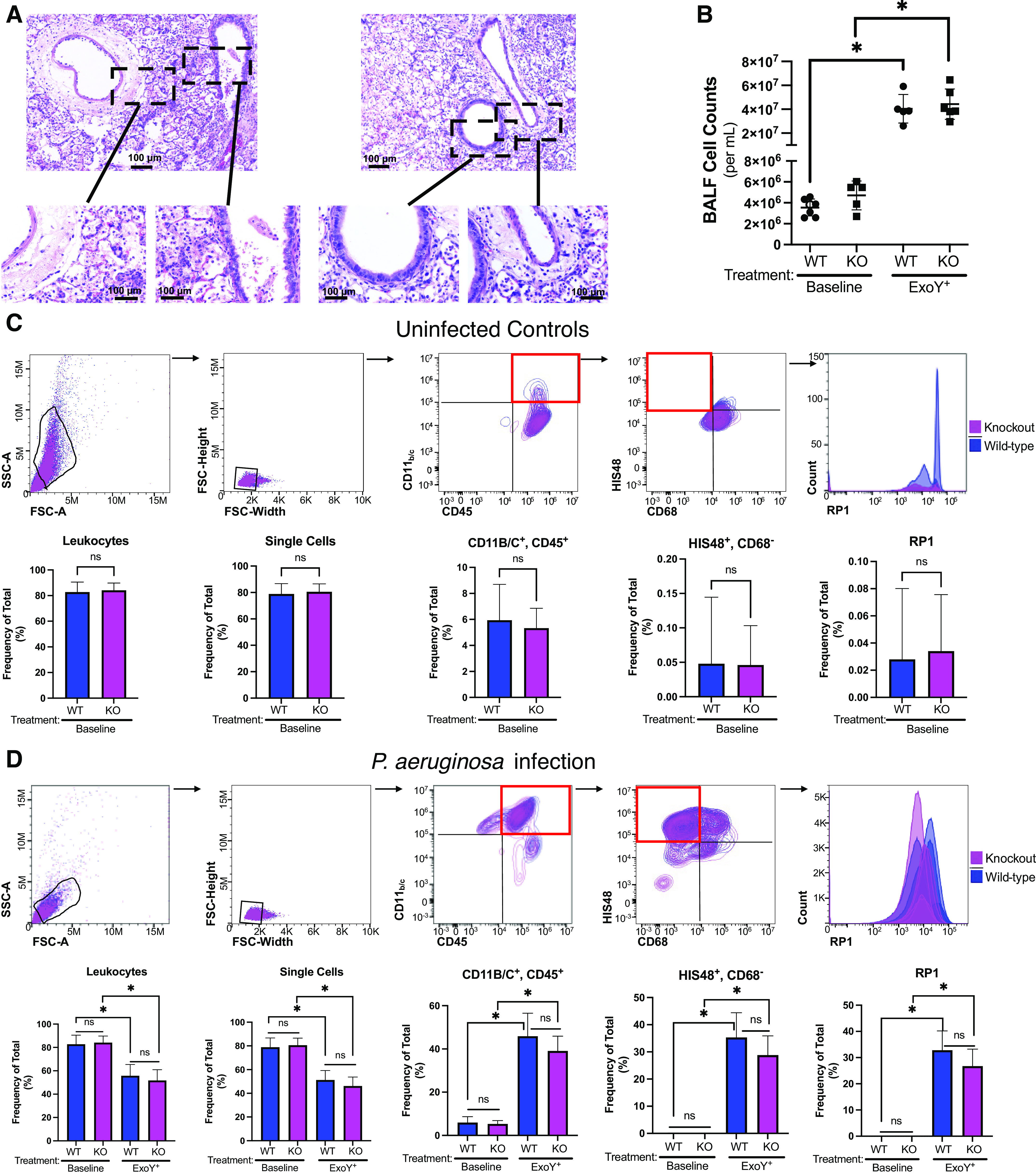
Infection evokes neutrophil recruitment to the airways. *A*: H&E staining of lung slices after ExoY^+^ infection shows inflammatory cell recruitment to the distal airways with consolidation and perivascular cuffing, consistent with pneumonia in both WT and KO rats. *B*: bronchoalveolar lavage fluid (BALF) was collected from WT and KO control and infected rats and analyzed using flow cytometry. Both the WT and KO infected rats had a higher number of cells in their BALF 48 h postinfection, indicating GSAP does not interfere with neutrophil recruitment. WT (*P* < 0.0001; *n* = 5–7) and KO (*P* < 0.0001; *n* = 5 or 6; main effect for genotype, *P* = ns, and for infection, *P* = ns) *C*: uninfected animals had similar numbers and types of cells in the airway. Cells retrieved from the BALF were selected based on their forward and side scatter and single cells selected from the forward scatter width and height. Single cells were labeled with a CD45 antibody targeting hematopoietic cells and a CD11b,c antibody targeting monocytes, granulocytes, macrophages, dendritic, and natural killer cells. CD45 and CD11b,c double positive cells were selected and then screened for cells within the population that interact with the macrophage antibody, CD68, and the granulocyte antibody, HIS48. The CD68 and HIS48 double positive cells were then labeled with the RP1 antibody targeting neutrophils. Few RP1 positive neutrophils were observed. *D*: neutrophils were recruited to the airways following infection. Forty-eight hours after infection, there was a significant increase in the number of CD45 and CD11b,c positive cells recovered from the airway. Within this population, there was also a significant increase in the number of CD68 low and HIS48 high cells, consistent with the presence of neutrophils in the airways. The CD68 low and HIS48 high cell population was positive for the neutrophil marker RP1. There was no difference in the number of neutrophils recovered in WT and KO animals (main effect for genotype, *P* = ns, and for infection, *P* ≤ 0.0001). Statistics were determined by two-way ANOVA with Tukey’s multiple comparisons test. Summary data are reported as means ± standard deviation. Closed symbols indicate male subjects. GSAP, gamma-secretase activating protein; H&E, hematoxylin-eosin; KO, knockout; WT, wild type. *Statistically significant difference.

To assess the pattern of acute lung injury, lungs were formalin-fixed at 25 cmH_2_O airway pressure, serially sectioned in hilar regions with consolidation, and hematoxylin-eosin (H&E) stained. Histology revealed a prominent leukocyte recruitment to the alveoli, a heterogeneous pattern of inflammation, alveolar consolidation and atelectasis, and perivascular fluid cuffing (Fig. 3*A*). This pathological pattern was consistent with pneumonia.

To assess whether GSAP contributes to the recruitment of neutrophils and monocytes to the airways, bronchoalveolar lavage fluid (BALF) was analyzed by flow cytometry. The heart and lungs were isolated en bloc and BALF was collected ex vivo. Similar numbers of cells were recovered in the BALF of wild-type and *Gsap* knockout rats at baseline and following infection, yet 10-fold more cells were recovered following infection (Fig. 3*B*). Cells recovered in the BALF were selected by forward and side scatter, and then single cells selected based on forward scatter width and height. A CD45 (hematopoietic cells) and CD11b,c (monocytes, macrophages, granulocytes) double high cell population was selected, and from that a CD68 (monocytes, macrophages) low and HIS48 high population was isolated. The CD68 low and HIS48 high population was then probed for the neutrophil marker, RP1. Whereas the BALF was nearly devoid of neutrophils at baseline (Fig. 3*C*), infection led to a significant increase in the number of RP1-positive cells (Fig. 3*D*). Although infection increased the number of neutrophils in the airways, there were no significant differences in the number of neutrophils detected in the airways of wild-type and knockout animals.

### GSAP Contributes to Disruption of the Alveolar-Capillary Barrier and Susceptibility to Myocardial Infarction during Infection

**Figure 4.**
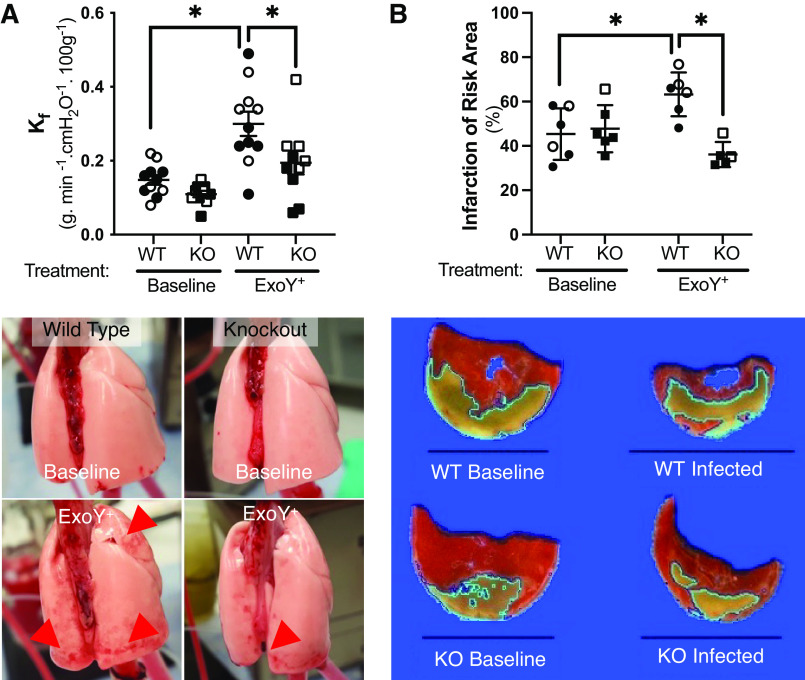
GSAP contributes to disruption of the alveolar-capillary barrier and susceptibility to myocardial ischemia-reperfusion injury following infection. *A*: ExoY^+^ was introduced into the trachea of the isolated perfused lung and filtration coefficient (*K*_f_) measured 4 h later. Infection increased *K*_f_ in the lungs from WT rats *(P* = 0.0003; *n* = 10 or 11), but did not increase *K*_f_ in the lungs from KO rats (*P* = ns; main effect for genotype, *P* ≤ 0.0001, and for infection, *P* = 0.0168). *K*_f_ in lungs from KO rats was significantly reduced when compared with wild-type rats postinfection (*P* = 0.0216; *n* = 10 or 11). *Top left*: summary data, means ± standard deviation, and the *bottom left* shows representative lungs before (baseline) and after infection (ExoY^+^). Red arrowheads highlight edematous lung regions. *B*: control and 48-h infected rats were assessed for myocardial susceptibility to 30 min of regional myocardial ischemia followed by 2 h of reperfusion. In uninfected rats, both WT and KO rats exhibited ∼45% infarction of the ischemic (risk) area and were not different from each other (*P* = ns between groups). Following infection, the percentage of the risk area that infarcted was increased by ∼30% in WT rats (*P* = 0.037; *n* = 5 or 6) and was increased compared with the KO rats following infection (*P* = 0.0028). Infarct size in the infected KO rats was not different from that in the uninfected KO rats (*P* = ns). A representative slice of the ischemic zone from a tetrazolium-stained heart from each of the four groups is shown below the summary data. The amount of infarct on each slice as determined by the ImageJ software is outlined. A black 1 cm scale line appears in each panel. Each slice was from a heart with an infarct size closest to the mean for each group. See the supplement for more details on the infarct sizing. Statistics were determined by two-way ANOVA with Tukey’s multiple comparisons test. Summary data are reported as means ± standard deviation. Open symbols reflect female subjects and closed symbols indicate male subjects. GSAP, gamma-secretase activating protein; KO, knockout; WT, wild type. *Statistically significant difference.

Results from blood oxygenation analyses suggested that *Gsap* knockout rats exhibit improved alveolar-capillary barrier integrity during infection. To test this idea, bacteria were added directly to the isolated perfused lung, and filtration coefficient was measured to evaluate vascular permeability. After an initial isogravimetric period and baseline filtration coefficient measurement, *P. aeruginosa* was introduced into the trachea and the filtration coefficient was measured 4 h later (Fig. 4*A*). Baseline filtration coefficient was not different in the lungs from wild-type and *Gsap* knockout rats. Whereas *P. aeruginosa* induced an increase in filtration coefficient in the wild-type lungs, this increase in permeability was not present in lungs from the *Gsap* knockout rats. Thus, GSAP contributes to disruption of the alveolar-capillary barrier during infection, even in the absence of a substantial neutrophil recruitment to the airways.

Susceptibility to cardiovascular disease, including myocardial infarction, stroke, and fatal coronary heart disease, is increased following pneumonia (34), although the mechanism(s) for this observation is unknown. We examined whether GSAP contributes to the severity of myocardial ischemia-reperfusion injury at baseline and following infection. The left anterior descending coronary artery was ligated for 30 min, followed by 2 h of reperfusion. Heart rate and blood pressure were not different between wild-type and knockout rats throughout the experiment, although both hemodynamic parameters gradually decreased over the 2.5-h time course (Supplemental Fig. S4). Quantification of infarcts revealed no differences between the ischemic zones in the hearts of wild-type and *Gsap* knockout rats (Fig. 4*B* and Supplemental Fig. S4). Thus, GSAP does not influence susceptibility to myocardial ischemia and reperfusion injury in uninfected rats.

In wild-type rats, pneumonia increased the infarcted myocardial region size by ∼30% compared with uninfected controls (Fig. 4*B* and Supplemental Fig. S4). However, pneumonia did not increase infarct size in hearts from the knockout rats. No postinfection differences in heart rate and blood pressure were seen in wild-type and *Gsap* knockout rats (Supplemental Fig. S4). Thus, infection reveals a novel GSAP-dependent mechanism of myocardial injury during ischemia and reperfusion.

### GSAP Limits Synaptic Strengthening and Suppresses Hyperexcitability of CA1 Neurons

**Figure 5.**
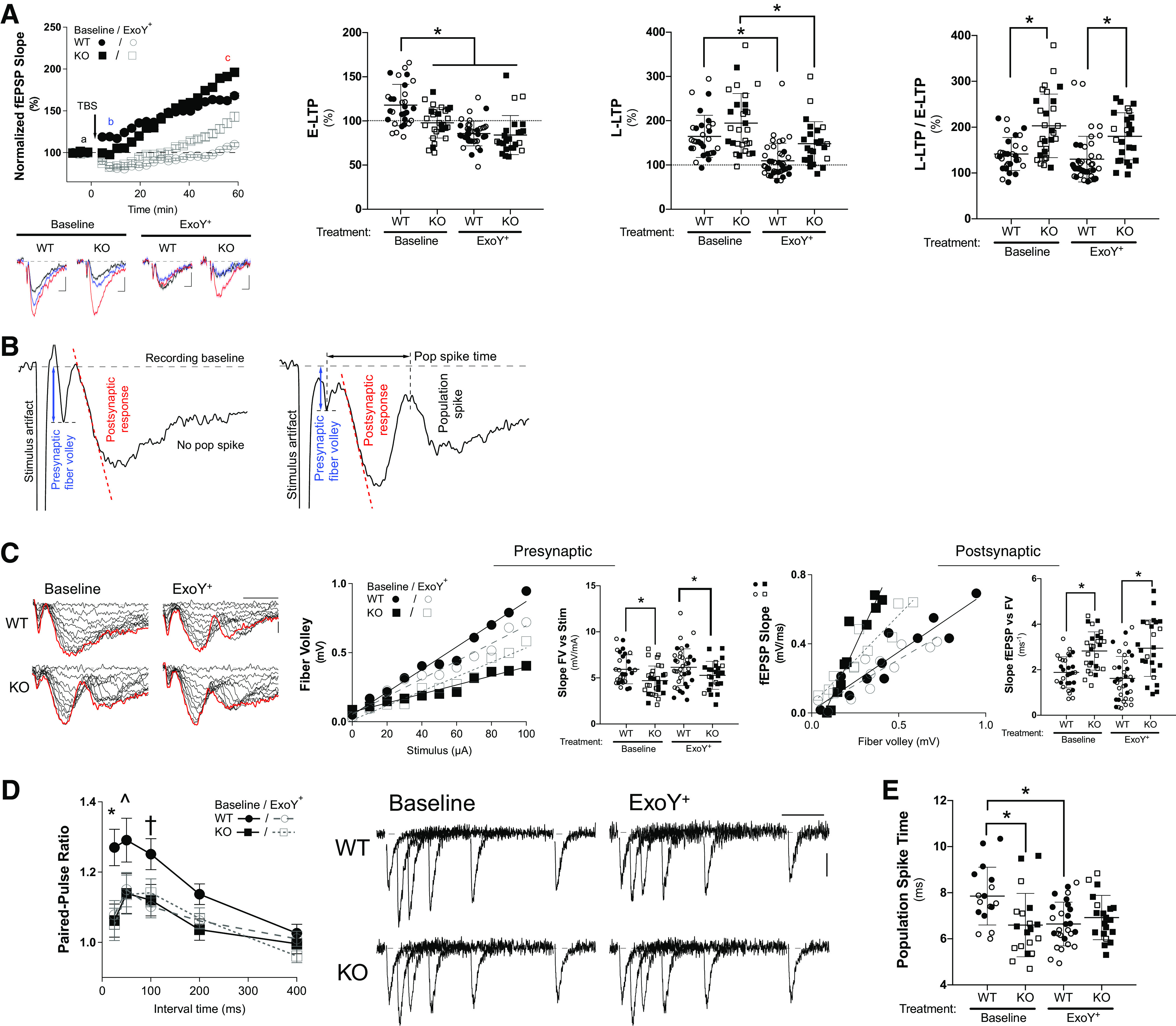
*Gsap* knockout rats exhibit elevated long-term potentiation, neurotransmitter release probability, and excitability. *A*, *left*: summary plot of long-term potentiation. Field excitatory postsynaptic potential (fEPSP) slopes were normalized to those before the theta-burst stimulation (TBS; delivered at *time 0*) and plotted against time (mean ± standard error of the mean). The *bottom* traces show averages of five representative traces obtained from time points “a” (−3-0 min; black), “b” (5–8 min; blue), and “c” (57–60 min; red) for uninfected baseline wild-type and *Gsap* knockout rats (WT, *N* = 30 slice recordings; KO, *N* = 29 slice recordings) and ExoY^+^ infected rats (WT, *N* = 40 recordings; KO, *N* = 25 recordings). Vertical and horizontal scale bars represent 0.1 mV and 5 ms, respectively. *Middle*: early LTP (E-LTP; 5–8 min post TBS) responses were plotted and compared. The response from uninfected WT was significantly higher than uninfected KO (*P* = 0.0016) and both WT (*P* < 0.0001) and KO (*P* < 0.0001) hippocampi following infection (main effect for genotype, *P* = ns, and for infection, *P* ≤ 0.0001). The late LTP (L-LTP; 57–60 min post TBS) plot shows that ExoY^+^ infection significantly reduced responses in both WT (*P* = 0.0024) and KO rats (*P* = 0.0179), and L-LTP trended toward a decrease in the WT rats after infection (*P* = 0.0791 vs. KO; main effect for genotype, *P* ≤ 0.0001, and for infection, *P* ≤ 0.0001). *Right*: ratio of the L-LTP vs. E-LTP plot shows that the amount of synaptic potentiation was higher in *Gsap* KO rats (WT vs. KO: before ExoY^+^: *P* = 0.0002 and after ExoY^+^: *P* = 0.01). Within the same genotype, there was no difference before and after infection (*P* = ns; main effect for genotype, *P* ≤ 0.0001, and for infection, *P* = ns). *B*: fiber volley was measured to assess the presynaptic depolarization-mediated axonal recruitment. The population spike, or “pop spike,” was measured as an indication of postsynaptic hyperexcitability. A representative fiber volley, postsynaptic response, and pop spike in hippocampi isolated from uninfected (*left*) and infected (*right*) animals is shown to illustrate these measurements. *C*, *Left*: representative elicited fEPSP traces in response to increasing stimulus intensity (0–100 µA with 10 µA increments). The red traces were elicited with 100 µA and highlight the appearance of population spike in WT only after ExoY^+^ infection and in KO with and without infection. From *left* to *right*, the first downward peak denotes presynaptic fiber volley elicited by Schaffer collateral electrical stimulation, and the next larger downward peak denotes the postsynaptic response; the third downward response indicates the population spike. Vertical and horizontal scale bars represent 0.3 mV and 5 ms, respectively. *Middle*: presynaptic fiber volley amplitudes were plotted against stimulus intensities, and the obtained slopes (FV vs. Stim) were compared across the four groups. KO hippocampi exhibited decreased presynaptic fiber volley amplitudes at baseline (WT, *N* = 30 recordings; KO, *N* = 30; *P* = 0.0246) and following ExoY^+^ infection (WT, *N* = 38; KO, *N* = 25; *P* = 0.041; main effect for genotype, *P* = 0.0004, and for infection, *P* = ns). *Right*: postsynaptic fEPSP slopes plotted against fiber volleys were line-fitted, and the slopes were compared. The postsynaptic responses were significantly different between WT and KO rats; baseline (WT vs. KO; *P* = 0.0027) and ExoY^+^ infected (WT vs. KO; *P* < 0.0001; main effect for genotype, *P* ≤ 0.0001, and for infection, *P* = ns). *D*, *left*: summary plot to study the presynaptic neurotransmitter release probability. A pair of stimuli were delivered to elicit fEPSPs. The paired-pulse ratios, determined from the amplitudes of second fEPSP over those of the first responses, were plotted against the interstimulus intervals (mean ± standard error of the mean). Uninfected WT showed significantly higher paired-pulse ratios at the 25 ms stimulus interval (WT vs. KO, *P* = 0.0196; WT vs. WT ExoY^+^, *P* = 0.0072; WT vs. KO ExoY^+^, *P* = 0.0037; main effect for genotype, *P* = ns, and for infection, *P* = 0.0248). *Right*: representative averages of five raw traces were overlaid, aligned, and normalized to the first fEPSP amplitude to show all evoked responses, and compared across the four groups. Vertical and horizontal scale bars represent 0.1 mV and 100 ms, respectively. *E*: population spike time was determined from the time between fiber volley peak (i.e., first downward peak in *B*) and the upward peak, between the postsynaptic response (second downward peak) and the population spike (third downward peak). When compared, the WT baseline was significantly different from the KO baseline (WT, *N* = 17 recordings; KO, *N* = 19; *P* = 0.0067), and from the ExoY^+^ infected WT (WT, *N* = 27; KO, *N* = 20; *P* = 0.0059). Statistics were determined by two-way ANOVA with Tukey’s multiple comparisons test. Summary data are reported as means ± standard deviation unless otherwise specified. Open symbols reflect female subjects and closed symbols indicate male subjects. GSAP, gamma-secretase activating protein; KO, knockout; WT, wild type. *Statistically significant difference.

Neurocognitive dysfunction is common during and in the aftermath of pneumonia (35). Thus, we examined the impact of pneumonia on long-term potentiation (LTP) at the Schaffer collateral synapses in hippocampi isolated from wild-type and *Gsap* knockout rats (Fig. 5*A*). When compared with controls, hippocampi obtained from the knockout rats exhibited a reduced synaptic response immediately after induction of LTP (at 5 min, E-LTP; WT: 118 ± 4.3% vs. KO: 97.9 ± 3.2%, *P* = 0.0005). However, LTP in the *Gsap* knockout hippocampi was potentiated with time, surpassing the LTP seen in the control animal hippocampi 30 min post-theta burst stimulation. The late LTP (at 60 min, L-LTP) trended higher in the knockout rats than it was in the control rats (WT: 165 ± 8.7% vs. KO: 195 ± 12.4%; *P* = 0.1159). The shift in synaptic strengthening from E-LTP to L-LTP appeared to differ. We assessed the amount of potentiation from E-LTP to L-LTP by examining their ratio. Results illustrate that *Gsap* knockout hippocampi had significantly higher L-LTP to E-LTP ratio than did the control hippocampi. Thus, the *Gsap* knockout hippocampi exhibited greater synaptic strengthening.

LTP was then measured 48 h after lung infection. Consistent with our prior studies (17, 18, 22), hippocampal E-LTP and L-LTP were abolished in the hippocampi of controls (Fig. 5*A*). LTP was also reduced in the hippocampi of *Gsap* knockout rats, however, the L-LTP was not abolished; the L-LTP response remained elevated when compared with wild-type hippocampi. Interestingly, the ratio of synaptic strengthening, i.e., the ratio of L-LTP to E-LTP, was higher in *Gsap* knockout hippocampi than it was in the wild-type hippocampi, with and without lung infection. Thus, the degree of synaptic potentiation was genotype-dependent and infection-independent.

To further explore the mechanisms by which GSAP regulates neuronal information processing, we quantified the input-output relationship from the field presynaptic fiber volley and postsynaptic response, respectively (Fig. 5*B*). Presynaptic fiber volley, indicative of depolarization-mediated axonal recruitment, was reduced in the knockout rats, suggesting that GSAP contributes to CA3 axonal depolarization and action potential firing induced by fast Na^+^ channels. However, for any given presynaptic depolarization, the excitatory postsynaptic response was higher in *Gsap* knockout rats (Fig. 5*B*), both at baseline and after lung infection, suggesting that GSAP governs coupling between presynaptic-to-postsynaptic (or input-output) neurotransmission. GSAP could increase presynaptic neurotransmission by modulating neurotransmitter release probability and it could decrease postsynaptic neurotransmission by reducing the neurotransmitter receptor density and/or signaling [i.e., *N*-methyl-d-aspartate (NMDA) and/or AMPA receptors], spine density, and/or the number of postsynaptic neurons recruited (17).

To investigate presynaptic neurotransmitter release probability, we recorded the paired-pulse ratio at 25, 50, 100, and 400 ms interstimulus intervals (Fig. 5*C*). Under baseline conditions, the paired-pulse ratio was lower throughout and significantly reduced at 50 ms in *Gsap* knockout versus the control hippocampi, indicating an increased neurotransmitter release probability in the knockout rats. Thus, whereas *Gsap* knockout hippocampi displayed a decrease in the presynaptic fiber volley (Fig. 5*B*), they possessed a potentiated neurotransmitter release probability and postsynaptic response with each stimulation. Lung infection reduced the paired-pulse ratio in control but not in *Gsap* knockout hippocampi. Thus, GSAP optimizes the efficiency of coupling between CA3 depolarization and neurotransmitter release at presynaptic boutons and postsynaptic densities, modulating the neurotransmitter release and receptor response, respectively.

We noted that the *Gsap* knockout CA1 neurons were hyperexcitable following Schaffer collateral stimulation under baseline conditions, and further, that both the wild type and *Gsap* knockout neurons were hyperexcitable following infection (Fig. 5*B*). We surveyed excitability quantifying population spikes from the dendritic field excitatory postsynaptic potential recordings (Fig. 5*D*). The time-to-population spike was calculated from the peak of the fiber volley to the groove between the two subsequent field potential peaks (i.e., the first peak indicates excitatory postsynaptic response, and the second peak denotes population spike or postsynaptic cell firing). The time-to-population spike was significantly faster in *Gsap* knockout than it was in control hippocampi under basal conditions (WT: 7.9 ± 0.30 ms vs. KO: 6.59 ± 0.32 ms, *P* = 0.0067), suggesting that the *Gsap* knockout postsynaptic neurons are hyperexcitable and more likely to fire action potentials. Lung infection increased the occurrence of population spikes in hippocampal slices prepared from both the control and *Gsap* knockout rats (WT: from 57% to 71% vs. KO: from 63% to 80%) and shortened the time-to-population spike in both control and knockout rats. Thus, GSAP dampens CA1 neuron excitability under basal conditions, and lung infection leads to hyperexcitability in both the wild-type and knockout CA1, suggesting that infection impairs GSAP function necessary to promote hyperexcitability.

## DISCUSSION

**Figure 6.**
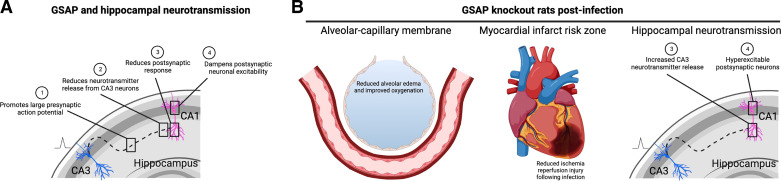
GSAP contributes to neurotransmission in the hippocampus under basal conditions and to lung, heart, and brain dysfunction following pneumonia. *A*: schematic highlighting the roles of GSAP in hippocampal neurotransmission. The collective effect of GSAP on neurotransmission is to facilitate the presynaptic action potential while dampening the postsynaptic response, thereby mitigating neuronal excitability. 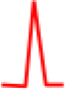
the presynaptic depolarizing current. *B*: the *Gsap* knockout rats exhibited improved lung and heart function following infection. In lung, the alveolar-capillary membrane was protected in the knockout animals. In heart, *Gsap* knockout animals were not protected from ischemia-reperfusion injury under baseline conditions, but did not exhibit the postinfection potentiation of the ischemia-reperfusion injury that was seen in wild-type animals. In the hippocampus, *Gsap* knockout rats exhibited increased CA3 neurotransmitter release and postsynaptic hyperexcitability following infection. GSAP, gamma-secretase activating protein. [Image created with BioRender.com and published with permission.]

Pneumonia is a common cause of end-organ dysfunction during, and in the aftermath of infection, yet the mechanism(s) responsible for end-organ dysfunction in this setting are poorly understood (36, 37). We have previously demonstrated that pneumonia due to clinical *P. aeruginosa* strains, PA103, and the ExoY^+^ mutant strain all elicits lung endothelial production of cytotoxic Aβ and tau, which induce end-organ dysfunction in the lung and brain (16–18, 38). These cytotoxins exhibit prion-like properties, meaning they are protease-resistant, heat-stable cytotoxins that are transmissible among cells and tissues and are self-propagating, i.e., they seed the production of new cytotoxic species (15, 39). Although we have addressed the mechanisms responsible for infection-induced cytotoxic tau production within the lung microcirculation (15, 17, 22, 30, 31), involvement of the molecular mechanisms underlying the amyloidogenic pathway have not been reported. Here, we examined whether bacterial pneumonia, due to ExoY^+^ infection, acts through GSAP necessary to injure the lung, heart, and brain. Consistent with this idea, *Gsap* knockout rats exhibited preservation of alveolar-capillary barrier integrity in the lung and reduced susceptibility to ischemia-reperfusion injury in the heart following infection. We found GSAP contributes to neural information processing under basal conditions, by dampening postsynaptic excitability at the Schaffer collateral synapses. However, GSAP did not appear to prevent the infection-dependent impairment of hippocampal long-term potentiation (Fig. 6). Therefore, GSAP contributes to pneumonia-induced end-organ dysfunction, particularly in the lung and heart.

Pneumonia is a direct cause of the acute respiratory distress syndrome (ARDS). The ARDS is characterized by pulmonary edema and refractory hypoxemia, especially in the early stages of the ARDS (40). Our studies illustrate a role for GSAP in both the pulmonary edema and hypoxemia that develop following infection. GSAP may contribute to disruption of the alveolar-capillary barrier: *1*) through an intracellular signaling function within the capillary endothelium and adjoining type I and type II pneumonocytes; *2*) by inhibiting Notch signaling; or, *3*) by promoting cytotoxic Aβ production. A signaling function of the 98-kDa holoprotein and its 82- and 16-kDa products is largely unexplored, aside from association of the 16-kDa product with gamma-secretase. Xu et al. (5) recently found the 16-kDa GSAP may interact with ∼80 proteins, including proteins involved in signal transduction, like protein phosphatases, and those involved in protein trafficking, lipid metabolism, and mitochondrial function. In particular, *Gsap* deletion improved mitochondrial oxygen consumption and promoted cellular survival. Future studies are needed to assess whether and how GSAP acutely impacts intracellular signaling events that disrupt the alveolar-capillary barrier and/or hinder repair.

Association of the 16-kDa GSAP with gamma-secretase increases gamma-secretase coupling to amyloid precursor protein, without negatively impacting the gamma-secretase interaction with Notch (1, 3, 8). It is unclear whether Notch signaling is enhanced in the alveolar-capillary membrane in the *Gsap* knockout rats. If so, then Notch signaling in the knockout rats may strengthen barrier integrity and promote repair (41, 42). Interestingly, gamma-secretase has been incriminated in lung repair; broad spectrum gamma-secretase inhibitors promote NOTCH signaling and increase the number of multiciliated cells (43).

Lung endothelium produces Aβ (15, 17, 23). Amyloid concentrations are increased in the bronchoalveolar lavage fluid and plasma of patients with pneumonia (18, 39), and here, we observed increased Aβ_42_ in the circulation of wild-type rats and increased Aβ_42/40_ ratios in both wild-type and GSAP KO rats postinfection. Nonetheless, the increase in circulating amyloids during infection cannot be specifically attributed to their production by lung endothelium. As Aβ_40_ and Aβ_42_ can be cytotoxic to the host (23), these infection-elicited amyloids may be an independent cause of injury to the alveolar-capillary barrier. Amyloids can also have antimicrobial properties (11–15). The posttranslational or structural modifications of Aβ_40_ and Aβ_42_ that confer these distinct functions are unknown, and it is unclear whether GSAP contributes to the production of antimicrobial versus cytotoxic Aβ variants, even in the absence of a significant difference in their circulating concentrations. Future studies are warranted to establish whether GSAP contributes to the pathologic and/or antimicrobial properties of amyloids, including Aβ_40_ and Aβ_42_.

Pneumonia increases the risk of myocardial infarction. The mechanisms placing these patients at risk for myocardial infarction have largely been attributed to inflammation and the prothrombotic, procoagulant state that accompanies infection (44, 45). The proinflammatory environment increases the risk of plaque rupture in susceptible patients resulting in ST-elevation myocardial infarction (STEMI), whereas hypoxemia, poor tissue perfusion, and increased myocardial oxygen demand promote the risk of demand ischemia leading to non-ST-elevation myocardial infarction (non-STEMI; 46). The current treatment for STEMI is to remove the coronary embolus by angioplasty or thrombolysis, but this can seldom be performed before a significant amount of the ischemic zone infarcts. A number of factors are thought to contribute to the cell death after a transient period of coronary ischemia, including apoptosis, necroptosis, mitochondrial-mediated necrosis, pyroptosis, ferroptosis, and autophagic cell death (47). Blockade of any of the above has been reported to make the heart more resistant to infarction. Similarly, activation of any of them will increase the infarct size. Infarcted myocardium cannot be regenerated so the strength of the heart, and thus the patient’s prognosis, is dependent on how much muscle is killed by infarction. We assessed susceptibility to infarction following ischemia-reperfusion injury at baseline and during infection. In wild-type rats, infection substantially increased the extent of myocardial infarction. Whereas GSAP was not incriminated in the mechanism of myocardial infarction in the uninfected heart, infection greatly increased the infarct size in our model, and surprisingly, that increase was found to be completely GSAP-dependent. Which, if any, of the aforementioned processes was involved in the increased vulnerability is unknown, but the fact that it is GSAP-dependent should aid in future studies seeking to resolve the mechanism.

GSAP is expressed in all brain regions, and via its influence on Aβ production, it has been incriminated in Alzheimer’s disease pathology (1). Yet, the role of GSAP in hippocampal information processing has not been determined. Our results support a central role for GSAP in four stages of information processing (Fig. 6). First, GSAP promotes a larger fiber volley response, meaning it contributes to a larger presynaptic action potential recruitment. Second, GSAP increases the paired-pulse ratio indicating a reduction in neurotransmitter release from CA3 neurons. Third, for any given presynaptic stimulation, GSAP reduces the postsynaptic response. Fourth, GSAP dampens postsynaptic neuronal hyperexcitability. Therefore, GSAP facilitates the presynaptic input while governing strength of the postsynaptic output, collectively increasing E-LTP and limiting L-LTP. This input-output tuning is consistent with the idea that distinct GSAP mechanisms control CA3 and CA1 activity, respectively. For example, GSAP’s interaction with gamma-secretase in CA3 neurons may optimize Aβ production and release, which in turn, provides negative feedback inhibition of both pre- and postsynaptic neurons. GSAP may also directly impact the postsynaptic response by orchestrating signaling events that occur through second messenger networks, trafficking of ion channels, and preservation of mitochondrial function, either in CA1 cells or other associated neuronal and/or glial cells (5). Future studies are needed to assess the Aβ-dependent and independent role of GSAP in neurotransmission.

Pneumonia acutely increases the risk for delirium, and it is a cause of incident dementia (25). Pneumonia promotes the accumulation of cytotoxic amyloids and tau within the cerebrospinal fluid within 48 h of infection (17, 22). These cytotoxic amyloid and tau variants impair hippocampal information processing, and over time, they reduce CA1 dendritic spine density (16, 18, 38). How infection is sensed, leading to the generation of cytotoxic Aβ variants, remains poorly understood. Here, we show that infection dramatically impairs neurotransmission in two ways (Fig. 6). First, while infection does not affect the presynaptic action potential it decreases the paired-pulse ratio characteristic of an increase in the neurotransmitter release probability from CA3 neurons. Second, it increases postsynaptic excitability. Collectively, this disruption in neurotransmission coincides with a reduction in LTP. In the absence of GSAP, hippocampal neurons have higher release probability and are more hyperexcitable, and lung infection does not further increase these responses. Future studies will be needed to assess whether postsynaptic hyperexcitability during infection is GSAP dependent. Nonetheless, infection-induced inhibition of LTP is mostly GSAP-independent, because the amount of inhibition in the presence or absence of GSAP expression was generally comparable. Thus, it is most likely that the infection-induced suppression of LTP is due to cytotoxic tau variants, as demonstrated in our prior study (22). The extent to which hyperexcitability contributes to suppressed LTP over time following infection remains to be determined. Although we did not perceive any overt phenotypical behavioral deviations in the novel *Gsap* knockout rat, the role of GSAP in animal behavior and learning and memory remains to be studied.

In conclusion, we provide evidence that bacterial pneumonia requires GSAP to orchestrate the molecular events underlying end-organ dysfunction in the lung and heart. Using this approach, our studies demonstrate GSAP plays a central role in hippocampal information processing, and plays an especially important role in suppressing neuronal hyperexcitability. Future studies are needed to determine the extent to which these GSAP-dependent mechanisms act through its regulation of the amyloidogenic pathway or result from other GSAP-dependent signaling events.

## DATA AVAILABILITY

Data will be made available upon reasonable request.

## SUPPLEMENTAL DATA

10.6084/m9.figshare.23304029.v2 Supplemental Tables S1 and S2 and Supplemental Figs. S1–S4: https://doi.org/10.6084/m9.figshare.23304029.v2.

## GRANTS

This work was supported by an American Heart Association predoctoral fellowship (PRE34381066; to M.S.G.); by Health and Human Services Grants HL66299 (to M.F.A., R.B., and T.S.), HL148069 (to M.F.A., R.B., and T.S.), HL140182 (to R.B., M.T.L., and T.S.), HL160988 (to J.Y.L. and T.S.), OD026560 (to A.M.G.), HL116264 (to A.M.G.), AG058780 (to A.R.N.), OD010944 (to M.F.A.), S10OD025089 (to M.F.A.), HL118334 (to J.P.A.), HL147512 (to S.V.), and HL007778 (to S.V.); by The Edward Via College of Osteopathic Medicine Grant VCOM-LA-BL (to S.V.), and by US Department of Defense (DOD) Grants W81XWH2110161 (to M.F.A.) and W81XWH2110669 (to M.A.).

## DISCLOSURES

No conflicts of interest, financial or otherwise, are declared by the authors.

## AUTHOR CONTRIBUTIONS

M.S.G., M.F.A., A.M.G., and T.S. conceived and designed research; M.S.G., M.F.A., J.Y.L., C.Z., X.-M.Y., S.V., V.V.P., J.B., L.A., R.B., M.T.L., and T.S. performed experiments; M.S.G., J.Y.L., C.Z., X.-M.Y., M.V.C., J.M.D., R.A.B., D.S., M.T.L., and T.S. analyzed data; M.S.G., M.F.A., A.M.G., J.Y.L., M.V.C., J.M.D., R.A.B., D.S., J.P.A., S.V., D.T.T., A.R.N., M.T.L., and T.S. interpreted results of experiments; M.S.G. and M.T.L. prepared figures; M.S.G., M.T.L., and T.S. drafted manuscript; M.S.G., M.F.A., J.Y.L., C.Z., M.V.C., J.M.D., R.A.B., J.P.A., D.W.F., S.V., D.T.T., M.T.L., and T.S. edited and revised manuscript; M.S.G., M.F.A., A.M.G., J.Y.L., C.Z., X.-M.Y., M.V.C., J.M.D., R.A.B., D.S., J.P.A., D.W.F., S.V., V.V.P., J.B., L.A., D.T.T., A.R.N., R.B. M.T.L., and T.S. approved final version of manuscript. 
